# A frailty index derived from a standardized comprehensive geriatric assessment predicts mortality and aged residential care admission

**DOI:** 10.1186/s12877-018-1016-8

**Published:** 2018-12-27

**Authors:** Rosie Burn, Ruth E. Hubbard, Richard J. Scrase, Rebecca K. Abey-Nesbit, Nancye M. Peel, Philip J. Schluter, Hamish A. Jamieson

**Affiliations:** 1 University of Otago, Christchurch, New Zealand; 2 Centre for Research in Geriatric Medicine, The University of Queensland, Brisbane, Australia; 3 Canterbury District health Board, Christchurch, New Zealand; 4 University of Canterbury – Te Whare Wānanga o Waitaha, Christchurch, New Zealand

**Keywords:** Aged care, Frailty index, Geriatric assessment, Community dwelling older people, Minimum data set

## Abstract

**Background:**

Frailty in older adults is a condition characterised by a loss or reduction in physiological reserve resulting in increased clinical vulnerability**.** However, evidence suggests that frailty may be modifiable, and identifying frail older people could help better target specific health care interventions and services.

**Methods:**

This was a regional longitudinal study to develop a frailty index for older adults living in Canterbury New Zealand. Participants included 5586 community dwelling older people that had an interRAI Minimum Data Set (MDS-HC) Home Care assessment completed between 2008 and 2012. The outcome measures were mortality and entry into aged residential care (ARC), after five years.

**Results:**

Participants were aged between 65 and 101 (mean age was 82 years). The five-year mortality rate, including those who entered ARC, for this cohort was 67.1% (*n* = 3747). The relationship between the frailty index and both mortality and entry into ARC was significant (*P* < 0.001). At five years, 25.1% (*n* = 98) of people with a baseline frailty of < 0.1 had died compared with 28.2% (*n* = 22) of those with a frailty index of ≥0.5 (FS 5). Furthermore, 43.7% (*n* = 171) of people with a frailty index of < 0.1 were still living at home compared to 2.6% (*n* = 2) of those with a frailty index of ≥0.5.

**Conclusion:**

A frailty index was created that predicts mortality, and admission into ARC. This index could help healthcare professionals and clinicians identify older people at risk of health decline and mortality, so that appropriate services and interventions may be put in place.

**Electronic supplementary material:**

The online version of this article (10.1186/s12877-018-1016-8) contains supplementary material, which is available to authorized users.

## Background

Frailty is considered a condition in older people characterised by a loss of physiological reserve, which causes increased clinical vulnerability [1–3].

Frail older adults are vulnerable to changes in their health status to the extent that any assault on the body, such as a minor infection, or a fall can have disproportionately negative outcomes [4, 5]. A measure of individual levels of frailty is needed to guide decision making, for example, when clinicians are considering treatment options in the aftermath of an acute episode or event, particularly as evidence suggests that frailty may be modifiable [6]. Identifying frail older adults would, therefore, help target specific health interventions and services needed to improve outcomes for this vulnerable cohort [1].

While there is a strong relationship between frailty and chronological age, frailty status is only one of many important factors that determine outcome; others include personal resources, social support and environmental factors, illness acuity and severity [7].

The measure of frailty has been considered using a phenotype model whereby the presence or absence of a pre defined set of five specific signs and symptoms are used to measure the degree of frailty of a specific individual [8]. Alternatively, frailty has been viewed from the perspective of an accumulation of deficits in the form of a frailty index [9]. A robust frailty index requires a significant number of individual items which are utilised to record deficit accumulation, and which are recorded as a score or index. This can also then be monitored in subsequent assessments to record the effectiveness or otherwise of specific interventions aimed at reducing an individual’s level of frailty [10].

A frailty index was recently developed using a standardised assessment of patients in acute care in Australia: the interRAI acute care (AC) instrument [6]. Using the methodology of Searle et al., 2008, [11] specific variables, common to all interRAI instruments, were identified within the assessment as potential health deficits. The interRAI-AC assessment used in that study is part of a validated interRAI suite of assessment tools, which also includes the Minimum Dataset-Homecare (MDS-HC) assessment [12–14]. The different interRAI tools are specifically designed so that core questions are the same and as a consequence a person’s health status can be tracked across different health settings using the appropriate interRAI assessment [15]. The MDS-HC assessment (recently updated and renamed as the interRAI Homecare assessment) is used in New Zealand to aid care of all older people living at home, who require publicly-funded community support or assessment for government-funded long-term residential care [16].

The MDS-HC has been demonstrated to be a valid and reliable electronic assessment tool. A detailed account of its successor, the interRAI-HC assessment instrument, has been described previously [16]. It includes 236 standardised questions analysing all aspects of an older person’s life [16]. Anyone requiring public funding for health services in New Zealand is required to undergo a needs assessment. An individual can be referred by health professionals such as their general practitioner, hospital-based professionals or community health workers. Generally, individuals requiring home care services are referred by a general practitioner or other health professional. A trained assessor visits the individual in their own home to conduct the home care assessment, the assessor spends time asking questions from the assessment, and items within the assessment include standardised responses with definitions and observational time periods. Assessors also refer to external health information such as patient records to ensure a complete and accurate picture of the individual’s health at time of assessment [15].

Using the recent Australian Frailty Index as a framework for our study, [6] we aimed to develop and validate a frailty score for the Canterbury, New Zealand community dwelling older people using local MDS-HC data.

## Methods

A regional longitudinal study was used to develop a frailty scale for older adults living in Canterbury, New Zealand. Outcome measures were mortality and entry into aged residential care (ARC) over a 5-year period. All data were anonymised. Ethics permission was granted by the New Zealand Ministry of Health and Disability Ethics Committee (14/STH/140).

Participants included 5586 community dwelling older people who had a MDS-HC assessment completed between 2008 and 2012, while living in the Canterbury province of New Zealand.

The MDS-HC is a standardised, geriatric, home care assessment consisting of over 200 questions, which are used to guide individual care planning. Assessments are conducted by trained assessors and each assessment is recorded electronically. Assessors visit the patient in person and ask questions, perform physical assessments and use up to date medical records.

Answers to 42 questions from the MDS-HC assessment were selected as variables in this study. The criteria for variable deficits from a recent Australian study were used in the selection of these variables [6]. Variables were recoded into deficits as described in the supplementary section. Most of the questions used in the frailty index were recorded on a binary scale of 0 or 1 where 1 represents the presence of the deficit and 0 represents the absence. For example, when a person’s memory was assessed, it was recorded as 0 = Memory is fine or 1 = Memory problem. These variables were directly translated into deficits. For ordinal and continuous variables, the answers were generally graded into deficits between 0 and 1, such as 0, 0.5, and 1. Thirty-eight of the 42 original variables were directly recoded into 38 potential deficits, while four variables had weightings on their deficit score. Three potential deficits were assigned to “behavioural symptoms”, “number of falls”, and “number of medications”, and 15 for “count of disease diagnosis”. This created a maximum score of 62. See Additional file 1 for the list of diseases in the interRAI-MDS.

The frailty index was calculated by summing the number of deficits recorded for a patient and dividing by the total number of possible deficits. This created a frailty index with a potential range from 0 to 1. In instances where data was missing, the frailty index was calculated with an appropriately reduced denominator, so for example if a person was missing data for one item the maximum score was reduced to 61. Anyone with a denominator of less than 30 was omitted from the study. In this study, there were no missing variables, the entire cohort had a denominator of 62.

For comparative purposes, the 0–1 frailty index was recoded into a 6-point frailty scale, where each person was allocated a score between 0 and 5 with 0 being the least frail and 5 being the frailest (Table 1). The scores on the scale correspond to the index as follows: Frailty Scale FS 0 having 0 ≤ FI < 0.1, FS 1 having 0.1 ≤ FI < 0.2, FS 2 having 0.2 ≤ FI < 0.3, FS 3 having 0.3 ≤ FI < 0.4, FS 4 having 0.4 ≤ FI < 0.5, and FS 5 having FI ≥ 0.5.

**Table 1:** Frailty index ranges

Failty index ranges	0-0.99	0.1-0.19	0.2-0.29	0.3-0.39	0.4-0.49	≥ 0.5
Frailty scale	0	1	2	3	4	5
Total^ (%)	391 (7.0)	1960 (35.1)	1993 (35.7)	877 (15.7)	287 (5.1)	78 (1.4)
Female^*^ (%)	198 (50.6)	1191 (60.8)	1265 (63.5)	551 (62.8)	178 (62)	45 (58)
Male^+^ (%)	193 (49.4)	769 (39.2)	728 (36.5)	326 (37.2)	109 (38)	33 (42)
Mean age	80.5	81.2	82.2	83.6	83.0	83.9

^^^Total people = 5586; ^*^total female = 3428 (61%); ^+^total male = 2158 (38.1%)

Dates of death were provided by the New Zealand Births, Deaths and Marriages dataset and matched to encrypted unique national identifier numbers. The NHI or National Health Index number is the unique person identifier used throughout the New Zealand public health system. Residential care entry date was obtained from the Contracted Care Payment System of the New Zealand Ministry of Health.

Normality of the results was tested using the Kolmogorov-Smirnov test. Kaplan–Meier curves were used to discern the relationships between the frailty scale and mortality and admission to residential care, after five years.

## Results

The total sample consisted of 5586 Canterbury District Health Board (CDHB), New Zealand, MDS-HC assessments. Participants were aged between 65 and 101 years, with a mean age of 82 years (SD 8.6 years). Most were females (3428; 61.3%) and European New Zealanders (4837, 86.6%). Individuals appeared to be cognitively healthy overall, however, over half of participants (2870, 51.4%) experienced a decline in ADLs and the majority have 2 or more disease diagnoses. The mean age increased with frailty (Table 1). Table 2 features a count of deficits used in creating the frailty index.

**Table 2:** Patient characteristics

Variable (Deficit score)	Frequency (%)
Cognitive skills for daily decision making	
0	3631 (65.0)
0.5	1536 (27.5)
1	419 (7.5)
Short Term memory	
0	3241 (58.0)
1	2345 (42.0)
Procedural memory	
0	4541 (81.3)
1	1045 (18.7)
Worsening of decision making	
0	4530 (81.1)
1	1056 (18.9)
Agitated or disoriented	
0	5262 (94.2)
1	324 (5.8)
Sudden or new onset/change in mental function	
0	5448 (97.5)
1	138 (2.5)
Making self understood	
0	4482 (80.2)
0.5	987 (17.7)
1	117 (2.1)
Ability to understand others	
0	4272 (76.5)
0.5	1169 (20.9)
1	145 (2.6)
Hearing	
0	2812 (50.3)
0.5	2676 (47.9)
1	98 (1.8)
Vision	
0	4047 (72.4)
0.5	953 (17.1)
1	586 (10.5)
Withdrawal from activities of interest	
0	4849 (86.8)
0.5	275 (4.9)
1	462 (8.3)
Repetitive anxious complaints, concerns	
0	4783 (85.6)
0.5	384 (6.9)
1	419 (7.5)
Sad, depressed	
0	4351 (77.9)
0.5	691 (12.4)
1	544 (9.7)
Behaviour Symptoms	
0	5280 (94.5)
1	203 (3.6)
2	72 (1.3)
3	31 (0.6)
Changes in behaviour symptoms	
0	5320 (95.2)
1	266 (4.8)
Changes in social functioning	
0	3116 (55.8)
0.5	1688 (30.2)
1	782 (14.0)
Bathing	
0	2530 (45.3)
0.5	1733 (31.0)
1	1323 (23.7)
Personal hygiene	
0	4411 (79.0)
0.5	340 (6.1)
1	835 (14.9)
Dressing upper body	
0	3922 (70.2)
0.5	1095 (19.6)
1	569 (10.2)
Dressing lower body	
0	3621 (64.8)
0.5	1209 (21.6)
1	756 (13.5)
Indoor mobility	
0	3362 (60.2)
0.5	2077 (37.2)
1	147 (2.6)
Outdoor mobility	
0	2565 (45.9)
0.5	2282 (40.9)
1	739 (13.2)
Transfer	
0	4848 (86.8)
0.5	519 (9.3)
1	219 (3.9)
Toilet use	
0	4749 (85.0)
0.5	173 (3.1)
1	664 (11.9)
Bed mobility	
0	5078 (90.9)
0.5	96 (1.7)
1	412 (7.4)
Eating	
0	5186 (92.8)
0.5	188 (3.4)
1	212 (3.8)
Mobility in home	
0	4835 (86.6)
0.5	339 (6.1)
1	412 (7.4)
Mode of locomotion	
0	3777 (67.6)
0.5	727 (13.0)
1	1082 (19.4)
Activities of daily living (ADL) decline	
0	2716 (48.6)
1	2870 (51.4)
Bladder continence	
0	3581 (64.1)
0.5	1165 (20.9)
1	840 (15.0)
Bowel continence	
0	4817 (86.2)
0.5	555 (9.9)
1	214 (3.8)
Disease diagnoses	
0	140 (2.5)
1	603 (10.8)
2	1081 (19.4)
3	1335 (23.9)
4	1070 (19.2)
5	666 (11.9)
6	365 (6.5)
7	201 (3.6)
8	77 (1.4)
9	32 (0.6)
10	9 (0.2)
11	7 (0.1)
Falls	
0	3297 (59.0)
1	2058 (36.8)
2	127 (2.3)
3	104 (1.9)
Unsteady gait	
0	2049 (36.7)
1	3537 (63.3)
Pain frequency	
0	1855 (33.2)
0.5	717 (12.8)
1	3014 (54.0)
Pain intensity	
0	1875 (33.6)
0.5	970 (17.4)
1	2741 (49.1)
Character of pain	
0	1910 (34.2)
0.5	1896 (33.9)
1	1780 (31.9)
Morbid obesity	
0	5477 (98.0)
1	109 (2.0)
Weight loss	
0	4683 (83.8)
1	903 (16.2)
Swallowing	
0	5057 (90.5)
0.5	514 (9.2)
1	15 (0.3)
Pressure ulcer	
0	5387 (96.4)
1	199 (3.6)
Medications	
0	115 (2.1)
1	1057 (18.9)
2	2269 (40.6)
3	2145 (38.4)
Congestive Heart Failure	
Not present	4352 (77.9)
Present	157 (2.8)
Present and treated	1077 (19.3)
Coronary Artery Disease	
Not present	4227 (75.7)
Present	223 (4.0)
Present and treated	1136 (20.3)
Hypertension	
Not present	2775 (49.7)
Present	351 (6.3)
Present and treated	2460 (44.0)
Alzheimer’s	
Not present	5375 (96.2)
Present	57 (1.0)
Present and treated	154 (2.8)
Dementia other than Alzheimer’s	
Not present	5279 (94.5)
Present	98 (1.8)
Present and treated	209 (3.7)
Parkinsonism	
Not present	5328 (95.4)
Present	33 (0.6)
Present and treated	225 (4.0)
Arthritis	
Not present	3243 (58.1)
Present	533 (9.5)
Present and treated	1810 (32.4)
Osteoporosis	
Not present	4427 (79.3)
Present	212 (3.8)
Present and treated	947 (17.0)
Any psychiatric diagnosis	
Not present	4726 (84.6)
Present	160 (2.9)
Present and treated	700 (12.5)
Urinary Tract Infection	
Not present	5232 (93.7)
Present	47 (0.8)
Present and treated	307 (5.5)
Cancer	
Not present	4792 (85.8)
Present	116 (2.1)
Present and treated	678 (12.1)
Diabetes	
Not present	4643 (83.1)
Present	166 (3.0)
Present and treated	777 (13.9)
Emphysema/COPD/Asthma	
Not present	4431 (79.3)
Present	173 (3.1)
Present and treated	982 (17.6)

**Fig. 1:**
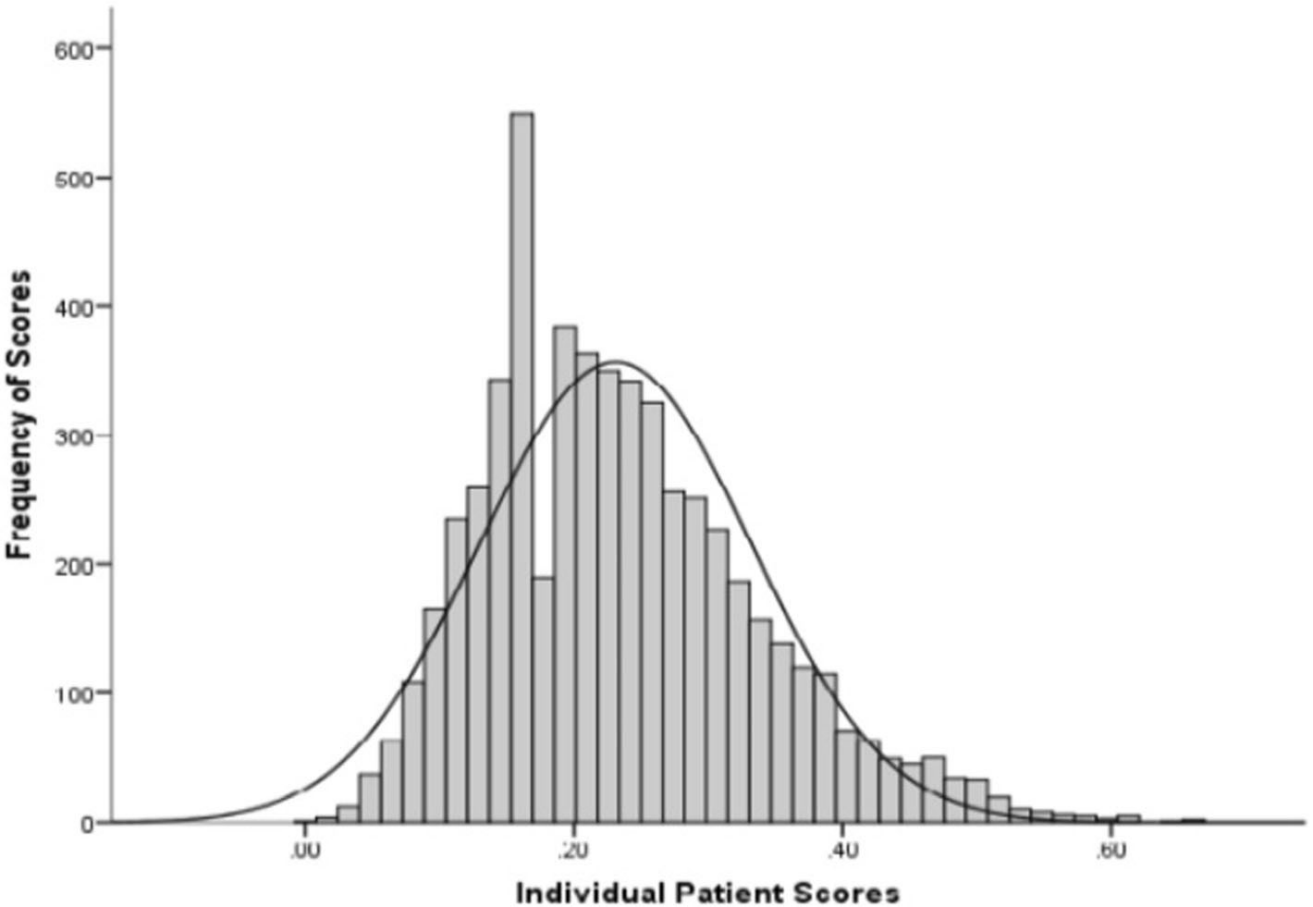
Distribution of Frailty Index

The frailty index had a mean of 0.27 (SD 0.12) and a range from 0.01 to 0.7. The index was not normally distributed (Fig. 1).

**Fig. 2:**
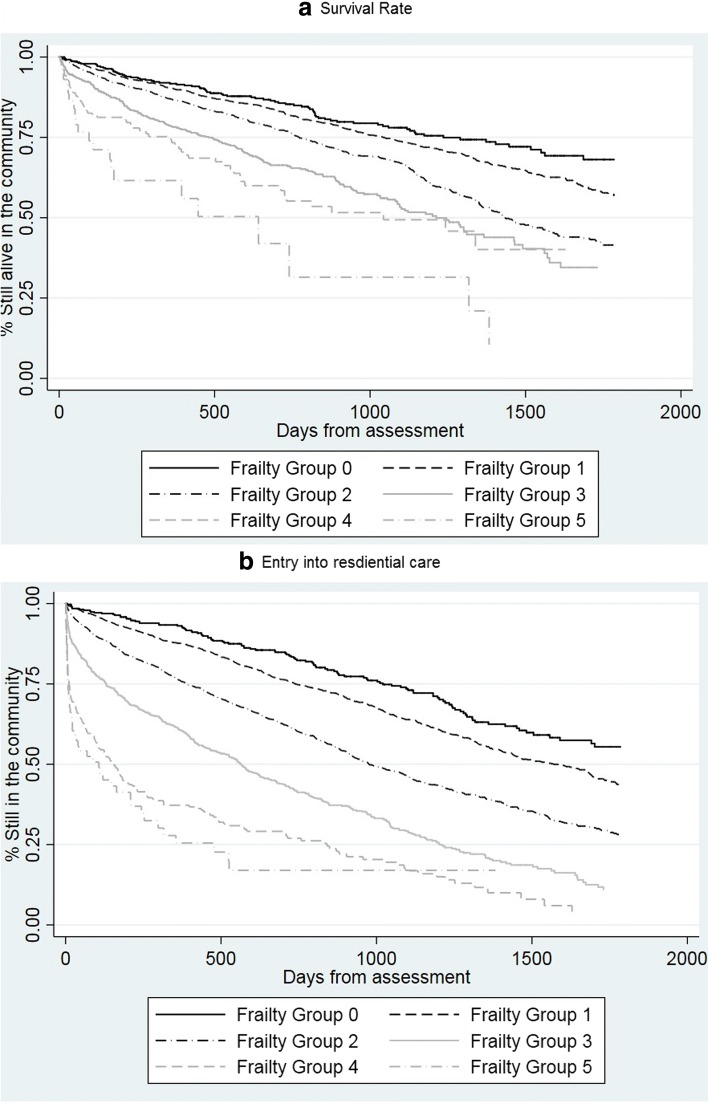
Survival Curves of **a** Mortality and Grouped Frailty, **b** Entry to ARC and Grouped Frailty

The five-year mortality rate was 67.1% (*n* = 3747). The relationship between the frailty score and mortality was significant (χ^2^ (5) = 332.2; *P* < 0.001). At five years, 25.1% (*n* = 98) of people with a baseline frailty of < 0.1 had died compared with 28.2% (*n* = 22) of those with a frailty index of ≥0.5 (FS 5),(Fig. 2a).

After five years 2670 (47.8%) of people had entered ARC. The relationship between the frailty index and admissions to residential care was also significant (χ^2^ (5) = 252.67; *P* < 0.001). At five years, 43.7% (*n* = 171) of people with a baseline frailty index of < 0.1 (FS 0) were still living at home, compared to 2.6% (n = 2) of those with a frailty index of ≥0.5 (FS 5),(Fig. 2b).

## Discussion

In this study we demonstrated that a frailty index developed from the interRAI MDS-HC assessment was significantly associated with five-year admission to residential care and mortality.

An Australian study used interRAI (AC) data from 1418 older adults presenting to acute hospitals in Queensland and Victoria formed the basis of our study [6].

This Australian study found a slightly higher mean frailty index (0.32; SD 0.14) than that of our work (0.27; SD 0.12) [6]. This difference could reflect the different settings of both studies: acute hospital care versus community dwelling. However, both studies had a similar dose response relationship for predicting mortality.

A recent large UK study, which calculated 36 deficits from pre-existing primary care health records, developed an electronic frailty index with four validated levels of frailty in over 900,000 older people [1]. Outcome measures over one-, three-, and five-year periods highlighted good predictive validity for emergency hospital admission, mortality, and nursing home admission. Survival rates were lower in our study than the UK study which could reflect our investigation’s focus on more frail and vulnerable members of a community dwelling population, rather than the general older population. The UK study demonstrated that risk of hospitalisation and length of hospital stay increased incrementally with the degree of frailty.

Another study of community dwelling older people contextualised frailty scores against clinical descriptors, [17] and found a mean FI of 0.27 for patients who were mildly frail, with limited dependence on others for instrumental activities of daily living. This is consistent with the findings of our study.

The MDS-HC assessment prepopulates the Change in Health, End Stage Disease (CHESS) score from 12 specific questions to create a 6 point score [18]. Although it is a valuable tool, the CHESS score is a measure of a person’s health instability rather than frailty and it focuses on recent changes in an individual’s health and level of function and does not include longer term indicators of frailty such as comorbidities and baseline activities of daily living (ADL) function. The larger number of potential deficits in our score allows for the identification of modifiable components which could be targeted to improve outcomes for individuals, such as exercise, nutrition programmes, and medication reviews to help reduce the effects of polypharmacy [7].

There are a number of other validated frailty scales, [1, 9, 19] but a significant advantage of our study and its utilisation of MDS-HC data, is that it allows for a comprehensive multi-dimensional perspective that aims to capture the complex nature of frailty without the need for further time consuming assessment. Furthermore, the fact that the interRAI suite of assessment tools are used over a number of different health settings means that individual levels of frailty using a standardised frailty index can be recorded both over time and across a variety of settings.

We acknowledge however, that this study utilised data from one region in New Zealand and that it may not be generalisable beyond this region. Additional work using this data set at a national level may be useful in identifying any ethnic differences in regard to frailty. Further work using New Zealand’s national MDS-HC dataset will assist in understanding this index’s generalisability including predicting other outcomes such as number and length of hospital admissions. We did not differentiate between frail individuals living at home or receiving daily care from those who live alone, and acknowledge that outcomes may have been poorer for the latter individuals.

## Conclusion

A frailty index was developed from Canterbury’s MDS-HC assessment data. From the index an easy to use scale was developed which could aid clinicians identify older people at risk of health decline and mortality. Frailty is not an inevitable part of ageing and nor should it be a barrier to interventions. It has the potential to be addressed and the individual’s outlook improved if it is identified early enough and the appropriate healthcare investigations, or services are initiated.

## Additional file

Additional file 1: RAN. This is a file containing information on how the frailty index was calculated. (DOCX 29 kb)
