# Supplementary material for: A frailty index derived from a standardized comprehensive geriatric assessment predicts mortality and aged residential care admission
Source: BMC Geriatr. 2018 Dec 27;18:319. doi: 10.1186/s12877-018-1016-8 (PMC6307300; doi:10.1186/s12877-018-1016-8)
Supplement: Supplementary file 1 — RAN. This is a file containing information on how the frailty index was calculated. (DOCX 29 kb) [file 12877_2018_1016_MOESM1_ESM.docx]

**Supplementary data**

The majority of the questions used in the frailty index were recorded on a binary scale of 0 or 1. For example, when assessing a patients memory it was recorded as 0 = Memory OK and 1 = Memory problem, or when asking whether the patient had an unsteady gait, it was answered as 0 = No and 1 = Yes. These variables were directly translated into deficits.

Recoding of other variables was more complicated. For variables where there was a three part response, such as when characterising pain it was answered as 0 = No pain, 1= localized –single pain, and 2 = multiple sites, and these responses were translated to 0, 0.5 and 1 respectively. Many variables were recorded on ordinal or continuous scales, which required are more complex recoding system where judgement was needed to determine the deficit contribution. For an ordinal scale example, when assessing ADL Self- performance, it was answered as: 0 = independent, 1 = setup help only, 2 = supervision, 3 = Limited assistance, 4 = Extensive assistance, 5 = maximal assistance, 6 = total dependence, 8 = activity did not occur (regardless of ability). For this example, it was recoded as 0-1 and 8 = 0, 2-3 = 0.5, and 4-6 = 1. There were some exceptions where 8 was representative the activity not occurring, for example when assessing primary mode of mobility, 8 was representative of the activity not occurring. In circumstances like this 8 was recoded as a deficit of 1. Variables recorded on a continuous scale, were recorded from 0 onwards in 1 point steps. For example hearing ability is recorded as: 0 = hears adequately, 1 = Minimal difficulty, 2 = Hears in special situations only, 3 = Highly impaired. For this example, the variables were recoded as 0=0, 1-2 = 0.5 and 3-4 = 1. To investigate behavioural symptoms, the following symptoms were selected: wandering, verbal abuse, physical abuse, socially inappropriate or disruptive behaviour, inappropriate sexual behaviour, and resisting care. Each symptom was answered as 0= did not occur in last three days, 1 = occurred but easily altered, and 3 = occurred but not easily altered. Deficit cut-offs were assigned based on the count of symptom points. Where 0 through 2 were directly translated to 0 to 2, while 3 or more was translated to 3.

Disease diagnoses was recorded based on the disease not being present = 0, present – not subject to focused treatments or monitoring by homecare professional = 1, and present – monitored or treated by homecare professional = 2. Each patient got one point per disease diagnosis whether disease was present = 1 or absent = 0, up to a maximum of 15 out of 28. Number of regular medications was determined by the count of medications and translated into cut-offs where: 0 medications =0, 1-4 medications = 1, 5-8 medications = 2, and 9 or more medications =3. Count of number of falls was done in the same way. In accordance with the well-defined methodology for FI derivation,(8, 16) when patients had missing data for one or more items, the total number of deficits considered for that patient was reduced; for instance, if a patient was missing data for one item the denominator was reduced to 61.

38 of the original variables considered were directly recoded into 38 potential deficits, while 4 variables had weightings on their deficit score. 3 potential deficits were assigned to “behavioural symptoms”, “number of falls”, and “number of medications”, and 15 for “count of disease diagnosis”.

| Domain | Instrument | (icode) | Question | Value Labels | InterRAI  Cut Offs | FI score | FI denominator  (running total) |
| --- | --- | --- | --- | --- | --- | --- | --- |
| Cognition | HC | B2a | Cognitive skills for daily decision making | 0 Independent  1 Modified independence  2 Minimally impaired  3 Moderately impaired  4 Severely impaired | 0  1 – 2  3 - 4 | 0  0.5  1 | 1 |
|  |  | B1a | Short term memory | 0 Memory OK  1 Memory problem | 0  1 | 0  1 | 2 |
|  |  | B1b | Procedural memory | 0 Memory OK  1 Memory problem | 0  1 | 0  1 | 3 |
|  |  | B2b | Worsening of decision making | 0 No  1 Yes | 0  1 | 0  1 | 4 |
|  |  | B3b | Agitated or disorientated | 0 No  1 Yes | 0  1 | 0  1 | 5 |
|  |  | B3a | Sudden or new onset/change in mental function | 0 No  1 Yes | 0  1 | 0  1 | 6 |
| Communication and Vision | HC | C2 | Making self understood | 0 Understood  1 Usually understood  2 Often understood  3 Sometimes understood  4 Rarely or never understood | 0  1 - 2  3 - 4 | 0  0.5  1 | 7 |
|  |  | C3 | Ability to understand others | 0 Understands  1 Usually understands  2 Often understands  3 Sometimes understands  4 Rarely or never understands | 0  1 - 2  3 - 4 | 0  0.5  1 | 8 |
|  |  | C1 | Hearing | 0 Adequate  1 Minimal difficulty  2 Hears in special situations only  3 Highly impaired | 0  1-2  3 | 0  0.5  1 | 9 |
|  |  | D1 | Vision | 0 Adequate  1 Impaired  2 Moderately impaired  3 Highly impaired  4 Severely impaired | 0  1  2 - 4 | 0  0.5  1 | 10 |

| Domain | Instrument | icode | Question | Value labels | InterRAI  Cut Offs | FI score | FI denominator  (running total) |
| --- | --- | --- | --- | --- | --- | --- | --- |
| Mood and behaviour | HC | E1h | Withdrawal from activities of interest | 0 Indicator not present in last 3 days  1 Exhibited on 1-2 of last 3 days  2 Exhibited daily in last 3 days | 0  1  2 | 0  0.5  1 | 11 |
|  |  | E1e | Repetitive anxious complaints, concerns | 0 Indicator not present in last 3 days  1 Exhibited on 1-2 of last 3 days  2 Exhibited daily in last 3 days | 0  1  2 | 0  0.5  1 | 12 |
|  |  | E1a | Sad, depressed | 0 Indicator not present in last 3 days  1 Exhibited on 1-2 of last 3 days  2 Exhibited daily in last 3 days | 0  1  2 | 0  0.5  1 | 13 |
|  |  | E3a-E3e | Behaviour symptoms: Count number present of the following: wandering, verbal abuse, physical abuse, socially inappropriate or disruptive behaviour, inappropriate sexual behaviour, resisting care. | 0 Did not occur in last 3 days  1 Occurred, easily altered  2 Occurred, not easily altered | 0  1  2  3 or more | 0  1  2  3 | 16 |
|  |  | E4 | Changes in behaviour symptoms | 0 No, or no change in behavioural symptoms or acceptance by family  1 Yes | 0  1 | 0  1 | 17 |
|  |  | F2 | Changes in Social Functioning | 0 No decline  1 Decline, not distressed  2 Decline, distressed | 0  1  2 | 0  0.5  1 | 18 |
| Functional Status | HC | H2j | Bathing | 0 Independent  1 Setup help only  2 Supervision- oversight, cueing  3 Limited assistance- guided manoeuvring of limbs  4 Extensive assistance- 1 person weight bearing support  5 Maximal assistance- 2 person weight bearing support  6 Total dependence  8 Activity did not occur (regardless of ability) | 0 – 1, 8  2 - 3  4 – 6 | 0  0.5  1 | 19 |
|  |  | H2i | Personal hygiene | 0 Independent  1 Setup help only  2 Supervision- oversight, cueing  3 Limited assistance- guided manoeuvring of limbs  4 Extensive assistance- 1 person weight bearing support  5 Maximal assistance- 2 person weight bearing support  6 Total dependence  8 Activity did not occur (regardless of ability) | 0–1, 8  2  3 – 6 | 0  0.5  1 | 20 |
|  |  | H2e | Dressing upper body | 0 Independent  1 Setup help only  2 Supervision- oversight, cueing  3 Limited assistance- guided manoeuvring of limbs  4 Extensive assistance- 1 person weight bearing support  5 Maximal assistance- 2 person weight bearing support  6 Total dependence  8 Activity did not occur (regardless of ability) | 0 – 1, 8  2 - 3  4 – 6 | 0  0.5  1 | 21 |
| Functional Status | HC | H2f | Dressing lower body | 0 Independent  1 Setup help only  2 Supervision- oversight, cueing  3 Limited assistance- guided manoeuvring of limbs  4 Extensive assistance- 1 person weight bearing support  5 Maximal assistance- 2 person weight bearing support  6 Total dependence  8 Activity did not occur (regardless of ability) | 0 – 1, 8  2 - 3  4 – 6 | 0  0.5  1 | 22 |
|  |  | H4a | Primary modes of mobility (Indoors) | 0 No Assistive device  1 Stick  2 Walker/crutch  3 Mobility scooter  4 Wheelchair  8 Activity did not occur | 0-1  2 - 3  4 – 6, 8 | 0  0.5  1 | 23 |
|  |  | H4b | Primary modes of mobility (Outdoors) | 0 No Assistive device  1 Stick  2 Walker/crutch  3 Mobility scooter  4 Wheelchair  8 Activity did not occur | 0-1  2 - 3  4 – 6, 8 | 0  0.5  1 | 24 |
|  |  | H2b | Transfer | 0 Independent  1 Setup help only  2 Supervision- oversight, cueing  3 Limited assistance- guided manoeuvring of limbs  4 Extensive assistance- 1 person weight bearing support  5 Maximal assistance- 2 person weight bearing support  6 Total dependence  8 Activity did not occur (regardless of ability) | 0 – 1, 8  2 – 3  4 – 6 | 0  0.5  1 | 25 |
|  |  | H2h | Toilet use | 0 Independent  1 Setup help only  2 Supervision- oversight, cueing  3 Limited assistance- guided manoeuvring of limbs  4 Extensive assistance- 1 person weight bearing support  5 Maximal assistance- 2 person weight bearing support  6 Total dependence  8 Activity did not occur (regardless of ability) | 0 – 1, 8  2  3 – 6 | 0  0.5  1 | 26 |
|  |  | H2a | Bed mobility | 0 Independent  1 Setup help only  2 Supervision- oversight, cueing  3 Limited assistance- guided manoeuvring of limbs  4 Extensive assistance- 1 person weight bearing support  5 Maximal assistance- 2 person weight bearing support  6 Total dependence  8 Activity did not occur (regardless of ability) | 0 – 1, 8  2  3 – 6 | 0  0.5  1 | 27 |
| Functional Status | HC | H2g | Eating | 0 Independent  1 Setup help only  2 Supervision- oversight, cueing  3 Limited assistance- guided manoeuvring of limbs  4 Extensive assistance- 1 person weight bearing support  5 Maximal assistance- 2 person weight bearing support  6 Total dependence  8 Activity did not occur (regardless of ability) | 0 – 1, 8  2  3 – 6 | 0  0.5  1 | 28 |
|  |  | H2c | Mobility in home | 0 Independent  1 Setup help only  2 Supervision- oversight, cueing  3 Limited assistance- guided manoeuvring of limbs  4 Extensive assistance- 1 person weight bearing support  5 Maximal assistance- 2 person weight bearing support  6 Total dependence  8 Activity did not occur (regardless of ability) | 0 – 1, 8  2  3 – 6 | 0  0.5  1 | 29 |
|  |  | H2d | Mode of locomotion | 0 Independent  1 Setup help only  2 Supervision- oversight, cueing  3 Limited assistance- guided manoeuvring of limbs  4 Extensive assistance- 1 person weight bearing support  5 Maximal assistance- 2 person weight bearing support  6 Total dependence  8 Activity did not occur (regardless of ability) | 0 – 1, 8  2  3 – 6 | 0  0.5  1 | 30 |
|  |  | H3 | ADL Decline | 0 Yes  1 No | 0  1 | 0  1 | 31 |
| Continence | HC | I1a | Bladder continence | 0 Continent  1 Continent with catheter  2 Usually continent  3 Occasionally incontinent  4 Frequently incontinent  5 Incontinent  8 Did not occur – no urine output from bladder | 0 – 1  2 – 3  4,5,8 | 0  0.5  1 | 32 |
|  |  | I3 | Bowel continence | 0 Continent  1 Continent with catheter  2 Usually continent  3 Occasionally incontinent  4 Frequently incontinent  5 Incontinent  8 Did not occur – no bowel movement during entire 7 day assessment | 0 – 1  2 – 3  4,5,8 | 0  0.5  1 | 33 |
| Disease | HC | J1a-J1ab | Count of disease diagnoses  (See syntax) | Count of explicit diagnoses  1 Present – not subject to focused treatment or monitoring by home care professional  2 Present – monitored or treated by home care professional | 1 point for each diagnosis up to 15 (Out of 28) | 1 - 15 | 48 |
| Health Conditions | HC | K5 | Falls Number of times fell in last 90 days (0-9) | Count of number of falls | 0  1-4  5-8  9 or more | 0  1  2  3 | 51 |
|  |  | K6a | Unsteady gait | 0 No  1 Yes | 0  1 | 0  1 | 52 |
| Pain  Health Conditions  (Cont.) | HC | K4a | Pain frequency | 0 No pain  1 Less than daily  2 Daily – one period  3. Daily – multiple periods | 0  1  2-3 | 0  0.5  1 | 53 |
|  |  | K4b | Pain intensity | 0 No pain  1 Mild  2 Moderate  3 Severe  4 Pain horrible/excruciating | 0  1  2 - 4 | 0  0.5  1 | 54 |
|  |  | K4d | Character of pain | 0 No pain  1 Localized – single site  2 Multiple sites | 0  1  2 | 0  0.5  1 | 55 |
| Oral and Nutritional Status | HC | L1c | Morbid obesity | 0 No  1 Yes | 0  1 | 0  1 | 56 |
|  |  | L1a | Weight loss of 5% or more in last 30 days, 10% or more in last 180 days | 0 No  1 Yes | 0  1 | 0  1 | 57 |
|  |  | L3 | Swallowing | 0 Normal  1 Requires Diet modification to swallow solid foods  2 Requires modification to swallow solid food and liquids  3 Combined oral and tube feeding  4 No oral intake | 0  1-2  3-4 | 0  0.5  1 | 58 |
| Skin | HC | N2a | Most severe pressure ulcer | 0 No pressure ulcer  1 Any area of persistent skin redness  2 Partial loss of skin layers  3 Deep craters in the skin  4 Breaks in skin exposing muscle or bone  5 Not codeable eg necrotic eschar predominant | 0  1 - 5 | 0  1 | 59 |
| Medications | HC | Q1 | Number of regular medications (no PRNs)  (see syntax) | Count of regular medications (no PRNs) | 0  1-4  5-8  9 or more | 0  1  2  3 | 62 |

Disease diagnoses included in the frailty index count:

1. Cerebrovascular accident
2. Renal Failure
3. Thyroid Disease
4. Congestive heart failure
5. Coronary artery disease
6. Hypertension
7. Irregularly irregular pulse
8. Peripheral vascular disease
9. Alzheimer’s
10. Dementia other than alzheimer’s
11. Head trauma
12. Hemiplegia/hemiparesis
13. Multiple sclerosis
14. Parkinsonism
15. Arthritis
16. Hip fracture
17. Other fractures (e.g. wrist, vertebral)
18. Osteoporosis
19. Cataract
20. Glaucoma
21. Any psychiatric diagnosis
22. HIV infection
23. Pneumonia
24. Tuberculosis
25. Urinary tract infection
26. Cancer
27. Diabetes
28. Emphysema/COPD/asthma
